# Glycan Profiling Identifies Chondroitin-4-sulfate as a Biomarker for Platinum Response and Therapeutic Target in Ovarian Cancer

**DOI:** 10.1101/2025.10.06.675352

**Published:** 2025-10-07

**Authors:** Erica J. Peterson, James D. Hampton, Ryan J. Weiss, Thomas M. Clausen, Ava R.S. Beaudin, Sharanya P. Deshmukh, Mikhail G. Dosmorov, Joseph B. McGee Turner, Amrita Basu, Elena Ethel Vidal-Calvo, Mads Daugard, Ali Salanti, Jennifer E. Koblinski, Larisa Litovchick, Nicholas P. Farrell

**Affiliations:** 1 Department of Internal Medicine, Virginia Commonwealth University; Richmond, VA, USA.; 2 Massey Cancer Center, Virginia Commonwealth University; Richmond, VA, USA.; 3 Bioplatinum Technologies LLC; Richmond, VA, USA.; 4 Department of Chemistry, Virginia Commonwealth University; Richmond, VA, USA.; 5 Department of Biochemistry and Molecular Biology, University of Georgia; Athens, GA, USA; 6 Complex Carbohydrate Research Center, University of Georgia; Athens, GA, USA.; 7 VAR2 Pharmaceuticals ApS, Copenhagen, Denmark; 8 Center for translational Medicine, Department of Immunology and Microbiology, University Copenhagen, Copenhagen, Denmark; 9 Department of Urologic Sciences, Faculty of Medicine, University of British Columbia, Vancouver, BC, Canada V5Z 1M9; 10 Vancouver Prostate Centre, Vancouver, BC, Canada V6H 3Z6; 11 Department of Pathology, Virginia Commonwealth University; Richmond, VA, USA.

## Abstract

For women with advanced ovarian cancer (OC), remission is typically achieved through surgery and combination chemotherapy, with duration largely dependent on tumor sensitivity to platinum-based drugs. Here, we show that tumor-associated glycosaminoglycans (GAGs) influence platinum drug efficacy in preclinical models of ovarian cancer. Due to the complexity of GAG biosynthesis and the involvement of multiple enzymes, traditional transcriptomic and proteomic approaches cannot accurately estimate their levels or correlation with patient response and survival. To address this, we quantitatively analyzed the full compositional profile of GAGs in OC patient-derived xenograft (PDX) models with known carboplatin sensitivity. Our results revealed a significant correlation between carboplatin resistance and high levels of the predominant GAG sequence, chondroitin-4-sulfate (C4S). Further investigation in cellular models demonstrated that high GAG expression reduces carboplatin uptake, DNA adduct formation, and tumor accumulation, whereas the opposite effect was observed for Triplatin, a GAG-targeting platinum agent. These trends were further validated in vivo, where treatment of OC PDX models with varying C4S levels confirmed that carboplatin efficacy decreases while Triplatin activity increases in tumors with high C4S expression. Based on these findings, we established a C4S cut-off score to predict tumor sensitivity, identifying a threshold above which tumors are likely to be carboplatin-resistant but Triplatin-sensitive. Analysis of patient tissue microarrays estimated that 40–83% of OC tumors, depending on subtype, exhibit high C4S expression. Collectively, these findings highlight the predictive power of C4S as a biomarker for platinum response and support the clinical evaluation of Triplatin as a targeted treatment for patients with carboplatin-resistant tumors expressing high levels of C4S.

## One Sentence Summary

Elevated chondroitin-4-sulfate in ovarian cancer results in resistance to carboplatin and sensitivity to Triplatin.

## INTRODUCTION

Ovarian cancer remains one of the most lethal gynecologic malignancies, ranking as the fifth leading cause of cancer-related mortality in women (1). The current standard-of-care involves cytoreductive surgery followed by combination chemotherapy with paclitaxel and a platinum-based agent, most commonly carboplatin. While the majority of patients achieve an initial response, disease relapse typically occurs, at which point the efficacy of subsequent platinum-based therapies is markedly reduced (2). Platinum-resistant ovarian cancer, defined as recurrence within six months of treatment, has a median survival of only 9–12 months, underscoring the need for novel therapeutic approaches. Moreover, some ovarian cancer subtypes, such as ovarian clear cell carcinoma, are inherently resistant to first-line platinum-based chemotherapy, making alternative treatments even more critical for these patients (3). In this work, we show that high chondroitin-4-sulfate (C4S) levels predict resistance of ovarian cancer tumors to carboplatin and conversely, sensitivity to the glycosaminoglycan (GAG)-targeting platinum agent, Triplatin/BBR3464 (4–8).

Platinum-based chemotherapeutics exert cytotoxic effects by forming DNA crosslinks, which interfere with DNA replication and transcription, ultimately triggering cell cycle arrest and apoptosis. However, platinum-resistant ovarian tumors frequently develop mechanisms that limit intracellular drug accumulation and improve the efficiency of DNA repair mechanisms/processes. These mechanisms include the downregulation of copper transporter 1 (CTR1), the primary cellular uptake transporter for cisplatin and carboplatin, as well as the upregulation of ATP7B, a copper-transporting ATPase that mediates platinum efflux (4,5). Additionally, increased activity of nucleotide excision repair pathways facilitates the efficient removal of platinum-DNA adducts, further reducing the drug’s cytotoxic potential. As a result, identifying biomarkers predictive of platinum resistance and developing targeted therapies capable of circumventing these resistance mechanisms remains a critical priority in ovarian cancer research.

**Fig. 1.**
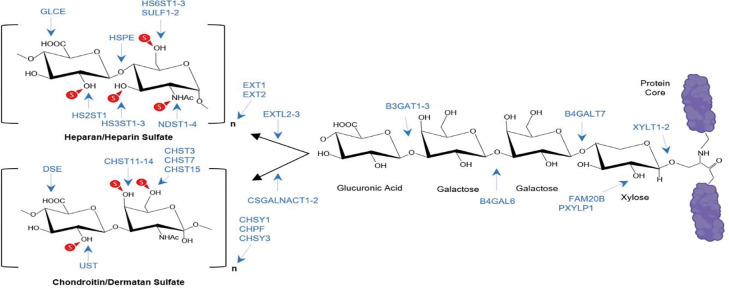
GAG levels are determined by expression of many enzymes Heparan sulfate or chondroitin /dermatan sulfate attachment sites in a proteoglycan protein core with a linker tetrasaccharide. Biosynthetic steps associated with the listed genes are indicated by arrows.

While the genetic and proteomic mechanisms underlying platinum resistance in ovarian cancer have been extensively characterized, the contribution of tumor-associated glycans, particularly GAGs, remains largely unexplored. GAGs are highly sulfated, linear polysaccharides localized at the cell surface and extracellular matrix, where they play critical roles in modulating tumor-stroma interactions and signal transduction. Unlike proteins and nucleic acids, GAG biosynthesis is non-template-driven and relies on a complex network of glycosyltransferases and sulfotransferases that regulate chain initiation, elongation and sulfation patterns (Fig. 1). Among the most abundant tumor-associated GAGs are chondroitin sulfate (CS) and heparan sulfate (HS), which influence growth factor signaling (e.g., FGF-FGFR axis), cell adhesion, angiogenesis, and immune evasion (11–21). Of particular interest is oncofetal chondroitin sulfate (ofCS), a tumor-specific GAG signature composed of unusually long chondroitin sulfate molecules carrying short islands of 6-O or non-sulfated CS in otherwise highly 4-O sulfated chains. ofCS has been shown to be expressed on the vast majority of tumors with very limited expression in normal tissues besides placental tissue and is thus an attractive therapeutic target for multiple malignancies (18, 22–31). However, its role in modulating platinum drug resistance has been poorly understood. While earlier published results indicated that the cationic nature of Triplatin contributes to its intrinsic charge-based interaction with GAGs, the inference that GAG also interferes with the cellular and tumor accumulation of carboplatin is entriely unexpected (32–34). These results expand our understanding of the antitumor mechanism of platinum agents and suggest approaches to the exploitation of GAG profile for precision medicine, warranting further investigation into how C4S expression influences chemotherapy response and whether targeting C4S could be exploited to overcome platinum resistance.

## RESULTS

### Subhead 1: Ovarian tumor GAG levels correlate with resistance to carboplatin

**Fig. 2.**
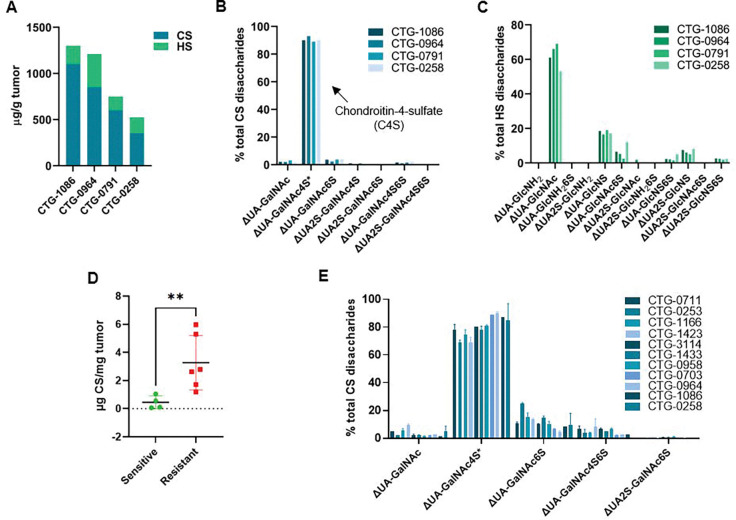
Carboplatin resistance correlates with chondroitin-4-sulfate levels in OC PDX models (**A**) Quantitative analysis of total GAG levels in OC PDXs using GRIL-MS (**B**) Compositional analysis of chondroitin sulfate in OC PDX using GRIL-MS (**C)** Compositional analysis of heparan sulfate in OC PDX using GRIL-MS (**D**) Quantitative analysis of the total GAG levels measured in 11 PDX models (2 tumors per model). PDX models were determined to be carboplatin-sensitive or resistant if the tumor growth inhibition (TGI) was >50% or <50%, respectively, in mice treated with 50 mg/kg i.p. carboplatin q7dx4. Tumor samples and drug screens provided by Champions Oncology. **p<0.01, student t-test. **(E)** Compositional analysis of chondroitin sulfate in OC PDX using

To investigate the relationship between GAG levels and platinum resistance in ovarian cancer, we performed quantitative and compositional glycan profiling of high-grade serous ovarian cancer patient-derived xenograft (PDX) tumors using glycan reductive isotope labeling–liquid chromatography/mass spectrometry (GRIL-LC/MS) (35). Analysis of four PDX models (Table 1) with known sensitivity to carboplatin revealed that chondroitin sulfate (CS) was the predominant GAG species (Figs. 2A and fig. S1). Notably, there was a high abundance of the monosulfated disaccharide, chondroitin-4-sulfate (C4S), but also low levels of chondroitin-6-sulfate (C6S) and unsulfated CS, altogether characteristics of the oncofetal-chondroitin sulfate disaccharide profile (Fig. 2B)(25, 27). In contrast, heparan sulfate (HS) was significantly less abundant than CS and exhibited greater structural heterogeneity, consisting of a mixture of unsulfated (>50%), monosulfated and disulfated disaccharides (Fig. 2C). Bulk RNA-seq profiling of genes involved in C4S biosynthesis—including select proteoglycan core proteins, glycosyltransferases responsible for chain initiation and elongation, and sulfotransferases mediating sulfation—showed concordance between transcriptional regulation of C4S biosynthesis and the glycan composition detected in each tumor. (fig. S2). To validate these findings, we expanded our analysis to additional ovarian cancer PDX models with known carboplatin sensitivity profiles, sourced from Champions Oncology. Notably, carboplatin-resistant models exhibited significantly higher C4S levels compared to carboplatin-sensitive models (Fig. 2D and E). These results suggest that elevated C4S expression correlates with platinum resistance in ovarian cancer, highlighting C4S as a potential biomarker for predicting chemoresistance and a targetable feature for therapeutic intervention. Further investigation is warranted to elucidate the mechanistic role of C4S in modulating drug response, including whether C4S-mediated sequestration or altered drug transport dynamics contribute to the reduced efficacy of carboplatin in resistant tumors.

### Subhead 2: Knockout of GAGs in OC cells affects platinum drug uptake and sensitivity

**Fig. 3.**
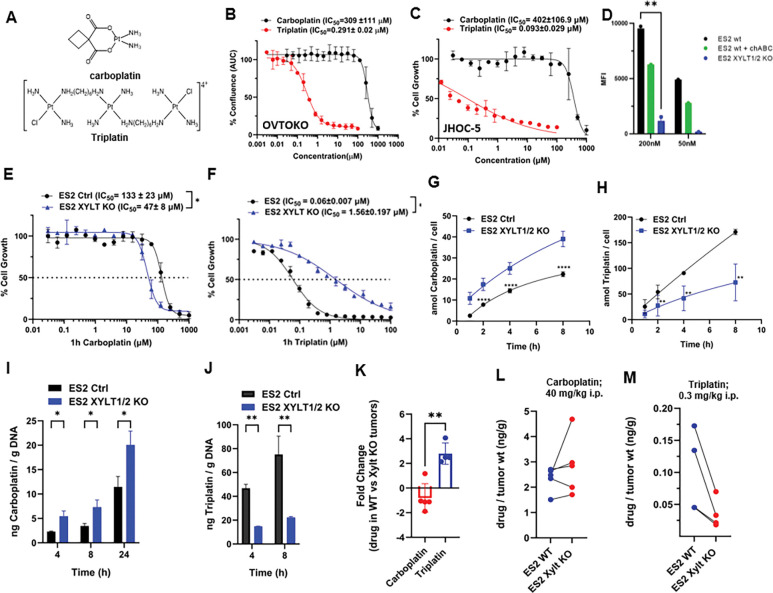
GAG levels influence the cytotoxicity, DNA platination and tumor uptake of carboplatin and Triplatin (**A**) Carboplatin and Triplatin chemical structures. (**B** and **C**) Cytotoxicity of carboplatin and Triplatin on human OVTOKO and JHOC5 cancer cell lines; 1h treatment. (**D**) Flow cytometry of 200 nM and 50 nM rVAR2-V5 binding to ES2 wt cells, ES2 wt cells + chABC treatment, or Xylt1/Xylt2 KO cells. (**E** and **F**) Cytotoxicity of carboplatin and Triplatin in ES2 wt and Xylt1/Xylt2 KO cell lines; 1h treatment. (**G** and **H**). Platinum cellular accumulation in ES2 wt and Xylt1/Xylt2 KO cells treated with 10 μM carboplatin or Triplatin for 1, 2, 4, and 8h. Platinum content was measured by inductively coupled plasma mass spectroscopy (ICP-MS) and normalized by number of cells. (**I** and **J**). Platinum-DNA adducts in ES2 wt and Xylt1/Xylt2 KO cells treated with 10 μM carboplatin or Triplatin 4, and 8h. (**K and M**) ES2-luc wt and Xylt1/Xylt2 KO tumors were implanted on the left and right flanks mice. Tumors were harvested after 24h treatment with 40 mg/kg i.p Carboplatin or 0.3 mg/kg i.p. Triplatin. Tumors were digested in nitric acid and platinum measured by ICP-MS.

To further explore the role of GAGs in platinum drug efficacy, we assessed the impact of GAG depletion on the cytotoxic activity of carboplatin and the GAG-targeting platinum agent Triplatin/BBR3464(36) (Fig. 3A). Triplatin is a highly charged, multinuclear platinum compound with a demonstrated affinity for sulfated GAGs (add ref). Using carboplatin-resistant ovarian clear cell carcinoma cell lines OVTOKO, JHOC-5, and ES2, we found that Triplatin exhibited significantly greater cytotoxicity compared to carboplatin (Fig. 3B,C). To determine whether GAG levels affect carboplatin activity, we generated GAG-deficient ES2 ovarian cancer cells using CRISPR-mediated knockout of xylosyltransferases XYLT1 and XYLT2, the enzymes responsible for the initial step in GAG biosynthesis. Effective GAG depletion was confirmed using flow cytometry with rVAR2-V5, a recombinant protein known to selectively bind tumor-specific oncofetal chondroitin sulfate (ofCS) (Fig. 3D)(18, 22–25, 27). In ES2 *XYLT* 1/2 KO cells, we observed enhanced carboplatin cytotoxicity, concurrent with an increase in platinum-DNA adduct formation and overall tumor accumulation of carboplatin. In contrast, Triplatin demonstrated reduced cytotoxicity, decreased cellular uptake, and less DNA adduct formation in GAG-deficient cells (Fig. 3E-M). These findings suggest that GAGs modulate platinum-based chemotherapy drug uptake, acting as a potential barrier to carboplatin accumulation while facilitating Triplatin retention and absorption into tumors. To validate these results, we extended our analysis to wild-type and GAG-deficient Chinese hamster ovary cells and observed a similar trend: carboplatin exhibited enhanced activity in GAG-deficient cells, whereas Triplatin showed reduced uptake and cytotoxicity (Fig. S3). As a proof-of-concept and to validate these hypotheses *in vivo*, we assessed the efficacy of carboplatin and Triplatin drugs in mice using an orthotopic ES2 ovarian cancer xenograft model. Mice treated with Triplatin exhibited a significant survival advantage compared to those treated with carboplatin (Fig. S4). These results establish a functional link between GAG levels and platinum drug response, underscoring the potential of C4S as both a predictive biomarker for carboplatin resistance and a therapeutic target for Triplatin-based intervention in ovarian cancer.

### Subhead 3: Carboplatin-resistant PDX with high C4S levels are selectively sensitive to Triplatin

**Fig. 4:**
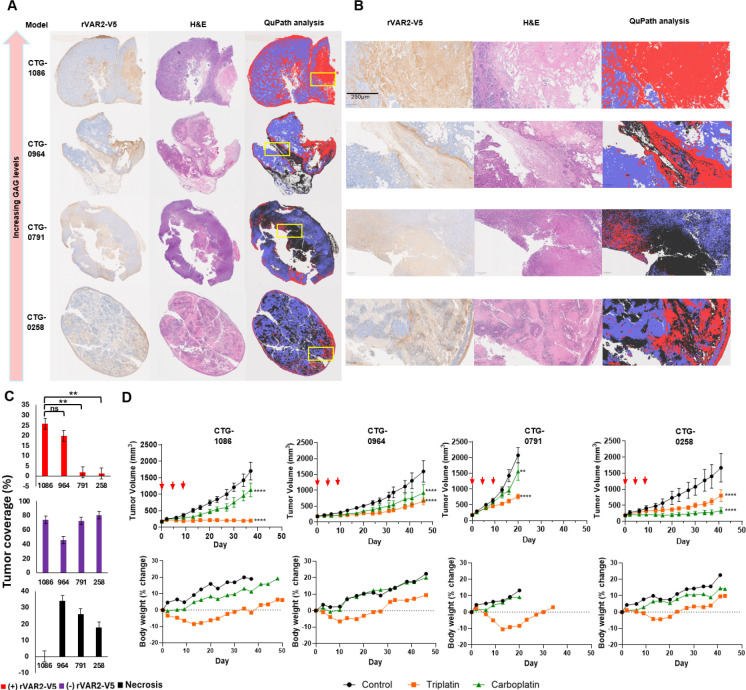
Triplatin inhibits tumor growth in carboplatin-resistant PDX expressing high levels of C4S (**A and B)** Representative images of rVAR2-V5 and H&E OC PDX staining and Qupath analysis. **(C)** Qupath segmentation of rVAR2-V5 (+), rVAR2-V5 (−), and necrotic tumor area in OC PDX models. **(D)** Sensitivity of OC PDX models to Triplatin and carboplatin. OC PDX models were treated i.p. with carboplatin (40 mg/kg) or Triplatin (0.3 mg/kg) on days 0, 4 and 8 (orange arrows). **p<0.01, ****p<0.0001, 2-way ANOVA, Tukey

Qupath digital pathology and whole slide analysis of OC PDX tumors from Table 1 stained with rVAR-V5 showed C4S staining levels consistent with the GRIL-LC/MS quantitative sequencing in Fig. 2 (Fig. 4A, B). The percentage of tumor area staining positive for C4S was highest in CTG-1086 (26%) and CTG-0964 (19.8%), whereas CTG-0791 (1.9%) and CTG-0258 (1.5%) exhibited minimal ofCS expression (Fig. 4C). Notably, the tumor-to-stroma ratio was inversely correlated with ofCS levels (Fig. S5), consistent with previous studies identifying ofCS as a major stromal constituent (27). To further validate the ofCS expression in those PDX tumors, we utilized newly developed antibodies targeting ofCS (27), which produced similar staining intensities and spatial distribution patterns across tumor samples (Fig. S6). Next, we evaluated the therapeutic response to carboplatin versus Triplatin in these PDX models and correlated it with ofCS levels. CTG-1086, which exhibited the highest ofCS levels, was highly sensitive to Triplatin but resistant to carboplatin. Conversely, CTG-0258, which had the lowest ofCS expression, was more sensitive to carboplatin and less responsive to Triplatin (Fig. 4D). Intermediate ofCS-expressing models (CTG-0964 and CTG-0791) demonstrated less difference between the efficacy of carboplatin and Triplatin (Fig. 4D). To further confirm this trend, we tested BBR3571, a structural analog of Triplatin with similar GAG-binding properties(37). BBR3571 exhibited comparable activity to Triplatin in high-ofCS-expressing tumors (Fig. S7), further supporting ofCS as a predictive biomarker for Triplatin efficacy. These findings establish that carboplatin-resistant ovarian cancer PDX models with high ofCS expression are sensitive to Triplatin. This suggests that ofCS enrichment in tumors may serve as a predictive biomarker for Triplatin responsiveness, highlighting the potential for ofCS -targeted patient stratification in future clinical trials.

### Subhead 4: Inherently platinum-resistant subtypes show prevalence of high C4S expression.

**Fig. 5:**
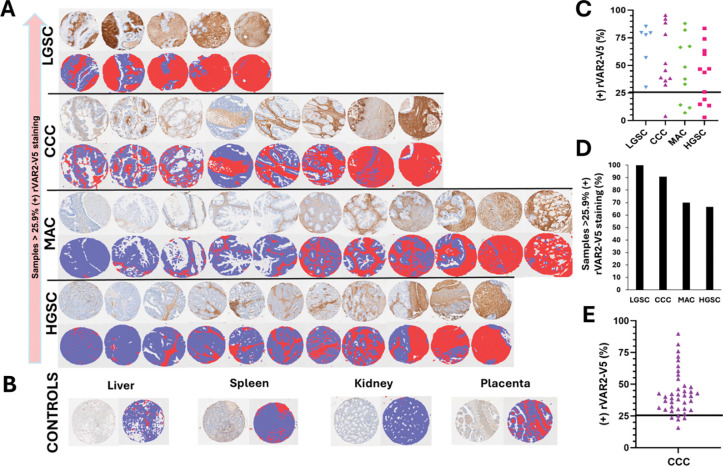
Abundance of ofCS in OC subtypes and normal tissues **(A)** UVA CHTN OC TMA; Representative samples of patient OC subtypes (clinical history unknown) and **(B)** normal tissues stained with rVAR2-V5 protein. **(C)** UVA CHTN OC TMA; Percentage of sample area staining positively for rVAR2-V5. Values are representative of the mean of 4 cores per patient sample. **(D)** UVA CHTN OC TMA; Percentage of TMA samples above the cut-off score. **(E)** UPenn CCC TMA samples; Percentage of sample area staining positively for rVAR2-V5.

To estimate the prevalence of C4S expression in ovarian cancer patients, we performed immunohistochemical staining of tissue microarrays (TMAs) using rVAR-V5. The OvCa2 TMA from the Cooperative Human Tissue Network at the University of Virginia includes representative cases of the major epithelial ovarian cancer histologic subtypes, including low-grade serous carcinoma (LGSC), clear cell carcinoma (CCC), mucinous adenocarcinoma (MAC), and high-grade serous carcinoma (HGSC) (Fig. 5A). Each tumor is sampled in quadruplicate with 0.6 mm tissue cores, allowing for reproducible and quantitative assessment of C4S expression across multiple tumor regions (Fig. 5B). To quantify C4S staining, we used Qupath segmentation and whole-slide analysis to determine the proportion score (PS), defined as the percentage of tumor area staining positive for C4S, as previously described in Fig. 4C. In this TMA cohort, we found that 100% of LGSC, 90% of CCC, 70% of MAC, and 65% of HGSC samples exhibited PS scores ≥ 26, a conservative threshold based on the correlation between C4S expression and Triplatin sensitivity (Fig. 5D). These findings suggest that tumors with PS ≥ 26 are more likely to be responsive to Triplatin than to carboplatin, reinforcing the potential for C4S-guided patient stratification in clinical applications.

To validate this trend in an independent cohort, we analyzed a second TMA from The BioTrust Collection at Penn Medicine, which consists of samples exclusively from patients with clear cell carcinoma, an inherently platinum-resistant ovarian cancer subtype. Notably, 92.5% of CCC cases exhibited PS scores ≥ 26, further supporting the strong association between high C4S expression and platinum-resistant ovarian tumor subtypes (Fig. 5E). These results establish that ofCS and thus C4S is highly expressed in ovarian cancer subtypes with intrinsic platinum resistance, including clear cell carcinoma, mucinous adenocarcinoma, and low-grade serous carcinoma. Given that these tumors exhibit poor response to carboplatin-based regimens, the high prevalence of C4S expression suggests that Triplatin may serve as a more effective therapeutic option for these patients.

## DISCUSSION

Our findings establish chondroitin-4-sulfate levels as a key determinant of carboplatin drug response in ovarian cancer, revealing a relationship between tumor-associated GAGs and platinum sensitivity. While the role of intracellular drug accumulation, DNA damage repair, and apoptotic signaling pathways in platinum resistance has been well established, this study identifies an extracellular component—tumor GAG levels—that modulates platinum drug efficacy at the tumor interface. Despite advances in targeted therapy and immunotherapy, platinum compounds continue to be the backbone of first line and subsequent treatment regimens, with nearly half of all cancer patients undergoing chemotherapy receiving a platinum agent (38). However, platinum resistance remains a major barrier to long-term treatment success, necessitating novel approaches to identify resistant tumors early and developing alternative therapeutic strategies.

We demonstrate that high levels of C4S correlate with carboplatin resistance but enhance sensitivity to Triplatin, a GAG-targeting platinum agent. This association is supported by quantitative glycan profiling, cellular and patient-derived xenograft models, and analysis of patient tumor tissue. Notably, low-grade serous and clear cell carcinomas exhibited the highest prevalence of C4S expression, providing a molecular basis for their well-documented poor response to standard-of-care platinum therapy. These results suggest that C4S expression could serve as a biomarker for platinum resistance and a companion diagnostic for selecting patients most likely to benefit from Triplatin treatment. Furthermore, our findings raise important mechanistic considerations regarding platinum drug uptake, sequestration, and activation in the tumor microenvironment. While the GAG-binding properties of Triplatin were anticipated based on prior research, the impact of C4S on carboplatin uptake, distribution, and efficacy was unexpected. Our results indicated that C4S may act as a physical or biochemical barrier, reducing carboplatin penetration and tumor accumulation. The prevailing model of platinum drug activation centers on the production of aqua species through substitution of leaving group (Cl−, CBDCA or oxalate in the case of oxaliplatin) within the intracellular environment, facilitating DNA crosslinking and cytotoxicity. However, our findings suggest that extracellular matrix components, particularly GAGs, may function as an overlooked “sink” of oxygen donors for platinum species, altering their local bioavailability and reactivity(39). This has significant implications for our fundamental understanding of platinum biology and tumor uptake, affecting pharmacokinetics and tumor penetration, warranting further investigation into the chemistry of platinum interactions with the extracellular matrix and its impact on drug activation and resistance.

While our study provides compelling evidence for the role of C4S in platinum drug response, several limitations must be acknowledged. First, tumor GAG levels may be heterogeneous within and across patient samples, meaning a larger-scale clinical dataset is required to fully establish C4S as a robust predictive marker for platinum sensitivity. Second, an outstanding question is whether C4S levels increase over the course of treatment as a resistance mechanism, or whether tumors with inherently high C4S expression are pre-disposed to platinum resistance. Longitudinal biopsy studies tracking C4S expression during treatment will be critical to addressing this question. Additionally, while we demonstrate that C4S influences carboplatin accumulation, other mechanisms such as copper transporter downregulation, efflux via ATP7B, and enhanced DNA repair pathways are known contributors to platinum resistance. Further studies are needed to determine whether C4S cooperates with or functions independently of these pathways. Lastly, the next step toward clinical application is to establish a clinically actionable threshold for C4S expression. Our study suggests a proportion score PS of ≥26% as a potential cutoff for Triplatin responsiveness, but further validation is required in a prospective clinical trial setting.

The findings presented here support the development of ofCS-directed treatment strategies in ovarian cancer, particularly for patients with recurrent platinum-resistant disease or those with inherently resistant histologic subtypes. Several key steps are necessary to translate these findings into clinical practice. Prospective clinical studies should evaluate C4S staining in biopsy specimens to determine whether C4S expression reliably predicts Triplatin sensitivity in patient cohorts. Additionally, Triplatin’s unique GAG-binding properties suggest a broader therapeutic application beyond ovarian cancer, particularly in other GAG-rich tumors such as pancreatic cancer, triple-negative breast cancer and bladder cancer, where similar resistance patterns have been observed(18, 22, 23, 27). If ofCS functions as a barrier to carboplatin entry, targeting ofCS, enzymatically or with competitive inhibitors, may enhance carboplatin efficacy. Another approach that ovarian cancer patients may benefit from is the use of ofCS-targeting modalities- such as antibody-drug conjugate or bispecific antibodies, which have demonstrated safe and potent anti-tumor activity in multiple animal models across diverse cancer types (22, 27, 40, 41). Future studies should investigate whether combining carboplatin with GAG-modulating agents can further optimize treatment outcomes. Given the growing emphasis on biomarker-driven treatment selection in oncology, incorporating ofCS profiling into precision medicine strategies may allow for more personalized and effective treatment regimens, particularly in platinum-resistant ovarian cancer. Future clinical studies will be essential to refining ofCS-based patient stratification, optimizing treatment selection, and developing next-generation platinum compounds that exploit tumor-specific GAG expression to overcome resistance.

## MATERIALS AND METHODS

### Study Design

The objective was to determine whether glycosaminoglycans (GAGs), particularly chondroitin-4-sulfate (C4S), modulate platinum response in ovarian cancer and whether C4S can serve as a predictive biomarker for Triplatin sensitivity. We combined quantitative glycomics of PDX tumors, in vitro perturbation of GAG biosynthesis, and in vivo efficacy studies in xenograft and PDX models, with IHC/TMA profiling and digital image analysis to define cut-points for potential patient stratification.

### Reagents

Triplatin was synthesized by published procedures (42). The malarial protein rVAR2 tagged with a V5 peptide (rVAR2-V5) was produced as described previously(22). Carboplatin (cat# PHR3417) and chondroitinase ABC were purchased from Sigma. Anti-V5 antibody (cat. # 96025) and anti-V5-Alexa Fluor 647 conjugate antibody (cat. # 451098) was purchased from ThermoFisher.

### Glycan profiling (Glycan Reductive Isotope Labeling Mass Spectroscopy)

Experiments were performed as previously described (43), with minor modifications. Flash-frozen PDX tumor tissues were pulverized and homogenized in protease digestion buffer. Samples were digested overnight at 37 °C with protease and centrifuged at 14,000 rpm for 20 minutes. The resulting supernatant containing released GAGs was applied to a DEAE ion exchange column, and bound GAGs were eluted with 2 M NaCl. Eluted GAGs were desalted using PD-10 Sephadex G-25 desalting columns (GE Healthcare), lyophilized, and resuspended in digestion buffer specific for chondroitin sulfate (CS) or heparan sulfate (HS). CS-containing samples were digested with a cocktail of chondroitinase ABC, while HS-containing samples were digested with a mixture of heparinase I, II, and III (New England Biolabs). Digestions were performed at 37 °C for 18 hours. Following digestion, samples were passed through 10,000 molecular weight cutoff filters to remove enzymes and undigested high-molecular-weight GAG chains. The filtrate containing disaccharide products was collected and dried under vacuum. The dried disaccharides were dissolved in aniline and derivatized by reductive amination using freshly prepared sodium cyanoborohydride in a dimethyl sulfoxide:acetic acid (7:3, v/v) mixture. Labeling reactions were incubated at 65 °C for 1 hour, followed by 16 hours at 37 °C. Samples were then dried in a speed vacuum concentrator at room temperature and submitted to the UCSD GlycoAnalytics Core for glycan analysis. Aniline-tagged disaccharides are separated on a C18 column using an ion pairing solvent mixture and analyzed by linear trap quadrupole mass spectrometry.

### Cell lines and culture conditions

CHO-K1 and CHO pgsA-745 were purchased from ATCC. CHO cell lines were cultured in DMEM-F12 media with 10% fetal bovine serum (FBS) and 1% penicillin/streptomycin. OVTOKO-*luc*, JHOC-5-*luc*, and ES2-*luc* cells were kindly provided by Dr. Ronny Drapkin at University of Pennsylvania. The ES2-luc were cultured in McCoy’s 5a (Invitrogen) with 10% FBS and 1% penicillin/streptomycin (Gibco). All cells were grown in a humidified atmosphere at 37°C with 5% CO_2_. Cells were routinely checked for mycoplasma contamination by PCR.

### CRISPR/Cas9 XYLT1/2 KO cell line generation

Cells lines were generated according to published procedures(44). GAG-deficient ES-2 cell lines were generated by transfecting the parental/wild-type ES2-luc cell line with either Cas9 plasmid and empty DNA backbone (control cells) or Cas9, *Xylt1*, and *Xylt2* gRNA plasmids (Knock-Out [KO] cells). Following transfection, cells were single cell sorted based upon GFP expression and allowed to grow in 96-well plates for 4 weeks, to allow for the clonal expansion of individual cell lines. Wild-type (wt), control, and KO cells were then screened *via* flow cytometry using GAG specific antibodies to identify GAG-deficient clones with XYLT1/2 KO and to confirm that control cell lines matched wt GAG levels. Following screening, cells were used for *in vitro* studies. Genomic alterations in KO cells were confirmed via cloning of Xylt1/2 PCR products and ligation into TOPO TA sequencing plasmid. Sequencing was carried out using Euro Fins Sanger Sequencing, M13F primer. PCR Primers – Xylt1fwd (gtttctctcccctcttctccag), Xylt1rev (atgtcacacttagggggctg), Xylt2fwd (cccagtgatgtgtctgcatc), Xylt2rev (gtatctccgtgtggtgggaag). Each cell line was sequenced 4 times for each gene to ensure accuracy.

### Cellular viability (MTT assay)

Cells were seeded in 96-well plates at a density of 1 × 10³ cells/well in 100 μL of complete growth medium and incubated for 24 h at 37 °C in an incubator with 5% CO. Cells were then treated with varying concentrations of drug or vehicle for 1 h. Following treatment, cells were washed once with PBS, and the drug-containing medium was replaced with fresh growth medium. Once the cells in control wells reached confluence, the media was replaced with 100 μL of MTT reagent (0.5 mg/mL in growth medium), and plates were incubated for 3 h at 37 °C. The MTT solution was then removed and replaced with 100 μL of DMSO to solubilize the formazan crystals. Plates were incubated for an additional hour at room temperature. Absorbance was measured at 570 nm using a microplate reader (BioTek Synergy), and background absorbance was subtracted from each reading. Cell viability was calculated relative to the untreated control wells, which were set to 100%. Data were analyzed using GraphPad Prism and reported as mean ± standard deviation from at least two independent experiments, each performed with four technical replicates.

### C4S quantification (Flow cytometry)

Cells are harvested using versene and washed twice in cold PBS. 1.5×10^6^ cells are resuspended in PBS containing 0.5% BSA and kept on ice throughout the procedure. The cells are incubated with 50 or 200nM rVAR2-V5 ofCS binding peptide for 30 minutes at 4°C. Binding was analyzed using a Fortessa flow cytometer (BD Biosciences) after washing twice with PBS, and a secondary incubation with 1:400 anti-V5 Tag Monoclonal Antibody, Alexa Fluor 647, (Invitrogen Cat. # 451098). For enzymatic GAG removal, ES2-wt cells were washed in PBS and incubated with 0.1 U/mL chondroitinase ABC in 2 ml buffer containing 50 mM Tris-HCl (pH 8.0), 60 mM sodium acetate and 0.02% BSA and for 30 min at room temperature. Cells were then washed twice with cold PBS/0.5% BSA and processed for rVAR2-V5 binding.

### Cellular platinum levels *in vitro* (ICP-MS)

Intracellular platinum content was determined as previously described (32), with minor modifications. Cells were seeded onto 100 mm tissue culture dishes and allowed to adhere for 24 h at 37 °C in an incubator with 5% CO. Cells were then treated with 10μM carboplatin or Triplatin for 0.5, 2, 4, or 8 hours. At each time point, cells were harvested by trypsinization, washed with PBS to remove extracellular drug and counted. Cell pellets were digested in trace-metal–grade nitric acid at room temperature for 72 hours. The digested samples were filtered through 0.22 μm PVDF filters and analyzed for platinum content using ICP-MS. Platinum concentrations were quantified against a standard curve generated using platinum standards of known concentration and normalized to total cell number. Data are presented as intracellular platinum per 10 cells.

### Cellular platinum-DNA adducts *in vitro* (ICP-MS)

Cells were seeded onto 100 mm dishes, allowed to attach for 24 h, and treated with 10 μM carboplatin or Triplatin. Following incubation times, cells were harvested via trypsinization. Cell pellets were washed repeatedly with PBS and DNA was extracted using the Qiagen DNA Easy Blood and Tissue kit. DNA samples were digested in trace-metal–grade nitric acid at room temperature for 72 hours. The digested samples were filtered through 0.22 μm PVDF filters and analyzed for platinum content using ICP-MS. Platinum concentrations were quantified against a standard curve generated using platinum standards of known concentration and normalized to the total grams of input DNA. Data are presented as intracellular platinum per gram DNA.

### Tumor platinum levels in vivo (ICP-MS)

For in vivo uptake (Fig. 3K–M), tumors were excised 24 h after a single dose of carboplatin (40 mg/kg i.p.) or Triplatin (0.3 mg/kg i.p.), weighed, minced, and digested in trace-metal–grade nitric acid (2 mL/100 mg tissue) for 72 h at RT. Digests were clarified (0.22 μm PVDF) and analyzed by ICP-MS against platinum standards. Data are reported as μg Pt per g wet tumor.

### PDX models and in vivo drug efficacy

Ovarian cancer PDX tumors with prior carboplatin sensitivity annotation were obtained via Champions Oncology for glycan profiling. The following drug efficacy studies were performed at Champions Oncology on four ovarian cancer PDX models (CTG-1086, CTG-0964, CTG-0791, CTG-0258). ~3mm^3^ PDX fragments were implanted subcutaneously into the flank of NSG mice (8–10 weeks old). Mice were randomized when tumor volume reached 100–200 mm^3^. Carboplatin (40 mg/kg i.p.), Triplatin (0.3mg/kg i.p.) or saline were administered on days 0,4, and 8 (q4dx3). Tumors were measured 2–3×/week by calipers; volume = (L×W^2^)/2. %TGI was computed at the indicated study day: %TGI = 100×[1 - (ΔT/ΔC)], where ΔT and ΔC are mean tumor volume changes from baseline in treated and control cohorts. Growth curves are means ± SEM

### ES2-luc xenograft studies

All animal procedures were approved by the Institutional Animal Care and Use Committee and conformed to NIH guidelines. Flank model (drug uptake). ES2-luc wt and XYLT1/2-KO cells (5×10 in 1:1 PBS:Matrigel; 100 μL) were injected into left/right flanks of female NSG mice. When tumors reached ~200mm^3^, mice received one dose of carboplatin (40 mg/kg) or Triplatin (0.3 mg/kg) i.p.; tumors were harvested 24 h later for ICP-MS (above).

### Tissue microarray cohorts

OvCa2 multi-histology TMA (University of Virginia CHTN; 0.6 mm cores, quadruplicate per case) and an independent clear cell carcinoma TMA (Penn Medicine BioTrust). Clinical histories were not available for analysis.

### Immunohistochemistry

C4S IHC staining using rVAR-V5. All tissues were fixed in 10% neutral buffered formalin for at least 5 days before paraffin embedding and sectioning. IHC staining for C4S using rVAR2- V5 peptide was performed in the VCU Cancer Mouse Models Core with the Leica Bond RX autostainer. Slides were baked at 60 °C for 60–120 min, deparaffinized, and rehydrated through graded ethanols to distilled water; antigen retrieval was not required for rVAR2 staining. rVAR2-V5 peptide was diluted in casein buffer to a final working concentration of 500 pM, and 150 μL was applied to each slide for 45 min at room temperature in a humidified chamber, followed by three washes in PBS or Leica wash buffer. Bound peptide was detected with mouse anti-V5 antibody (R960–25, ThermoFisher; 1:700 in casein buffer, 45 min, room temperature), followed by Leica post-primary rabbit anti-mouse antibody (8 min) and goat anti-rabbit HRP conjugate (8 min), with PBS washes between each step. Slides were developed with 3,3′-diaminobenzidine (DAB, Leica kit) for 10 min, rinsed in water, counterstained with hematoxylin, dehydrated through graded ethanols, cleared in xylene, and mounted. Positive controls included previously validated tumors with high and low C4S expression, and negative controls omitted rVAR2-V5 to test for background staining from the anti-V5 antibody alone. Stained slides were scanned at 20× magnification using a Vectra Polaris whole-slide scanner and analyzed in QuPath for quantitative assessment of rVAR2-V5 positivity.

### Digital pathology and image analysis (Qupath)

The proportion score (PS) was defined as % tumor area that is rVAR2-V5–positive above a fixed DAB OD threshold determined on control tissue and applied batch-wise. For each case, PS was averaged over four cores. Necrotic regions and artifacts were excluded manually. Cut-point; a conservative cut-point of PS ≥ 26 was selected a priori from PDX-anchored correlation analyses (linking rVAR2-V5 area fraction to Triplatin-carboplatin response separation) and then applied to TMAs to estimate prevalence by subtype.

### Statistical analysis

Statistically significant differences were tested using assay specific tests as indicated in the figure legends. One-way or two-way analysis of variances (ANOVA) was performed to determine significant differences among the control and treatment groups. Tukey’s multiple comparisons tests (for one-way ANOVA) or Dunnett’s multiple comparison test (for two-way ANOVA) were performed to for comparison across groups. Unpaired t-tests were performed to compare 2 groups. Kaplan-Meier survival plots were generated and changes in survival were analyzed by the log-rank test. All analyses were calculated in GraphPad Prism 10 software and p values < 0.05 were considered significant.

## Supplementary Material

Supplement 1

## Figures and Tables

**Table 1.** PDX characteristics, patient treatments, and platinum sensitivity

Ovarian Cancer PDX			Patient Treatments (pre-collection)			Carboplatin Sensitivity*	
ID	Site	Diagnosis	Drugs	Outcome	Duration	Dose (mg/kg); Sch.	%TGI (dT/dC)
CTG-1086	Sm. bowel	Recurrent	Carbo/Paclitaxel/Bevac.	Responded	12	50 i.p.; q7d X4	−24
CTG-0964	Peritoneum	Recurrent	Carbo/Paclitaxel	Responded	24	50 i.p.; q7d X4	29
CTG-0791	Abdomen	First	Carbo/Paclitaxel**	No response	0	50 i.p.; q7d X4	17
CTG-0258	Lymph	Unknown	Unknown	Unknown	Unknown	50 i.p.; q7d X4	105

## Data Availability

All data are available in the main text or the supplementary materials.

## References

[1] Siegel R. L., Giaquinto A. N., Jemal A., Cancer statistics, 2024. CA. Cancer J. Clin. 74, 12–49 (2024). 38230766 10.3322/caac.21820

[2] Lheureux S., Braunstein M., Oza A. M., Epithelial ovarian cancer: Evolution of management in the era of precision medicine. CA. Cancer J. Clin. 69, 280–304 (2019). 31099893 10.3322/caac.21559

[3] Shoji T., Tatsuki S., Abe M., Tomabechi H., Takatori E., Kaido Y., Nagasawa T., Kagabu M., Baba T., Itamochi H., Novel Therapeutic Strategies for Refractory Ovarian Cancers: Clear Cell and Mucinous Carcinomas. Cancers 13, 6120 (2021). 34885229 10.3390/cancers13236120 PMC8656608

[4] Manzotti C., Pratesi G., Menta E., Di Domenico R., Cavalletti E., Fiebig H. H., Kelland L. R., Farrell N., Polizzi D., Supino R., Pezzoni G., Zunino F., BBR 3464: a novel triplatinum complex, exhibiting a preclinical profile of antitumor efficacy different from cisplatin. Clin. Cancer Res. Off. J. Am. Assoc. Cancer Res. 6, 2626–34 (2000). 10914703

[5] Manzotti C., Pratesi G., Menta E., Di Domenico R., Cavalletti E., Fiebig H. H., Kelland L. R., Farrell N., Polizzi D., Supino R., Pezzoni G., Zunino F., BBR 3464: A Novel Triplatinum Complex, Exhibiting a Preclinical Profile of Antitumor Efficacy Different from Cisplatin1. Clin. Cancer Res. 6, 2626–2634 (2000). 10914703

[6] Sessa C., Capri G., Gianni L., Peccatori F., Grasselli G., Bauer J., Zucchetti M., Viganò L., Gatti A., Minoia C., Liati P., Van den Bosch S., Bernareggi A., Camboni G., Marsoni S., Clinical and pharmacological phase I study with accelerated titration design of a daily times five schedule of BBR3464, a novel cationic triplatinum complex. Ann. Oncol. 11, 977–984 (2000). 11038034 10.1023/a:1008302309734

[7] Gourley C., Cassidy J., Edwards C., Samuel L., Bisset D., Camboni G., Young A., Boyle D., Jodrell D., A phase I study of the trinuclear platinum compound, BBR 3464, in combination with protracted venous infusional 5-fluorouracil in patients with advanced cancer. Cancer Chemother Pharmacol 53, 95–101 (2004). 14605864 10.1007/s00280-003-0721-x

[8] Calvert A. H., Thomas H., Colombo N., Gore M., Earl H., Sena L., Camboni G., Liati P., Sessa C., Phase II clinical study of BBR 3464, a novel, bifunctional platinum analogue, in patients with advanced ovarian cancer. Eur. J. Cancer 37, S260 (2001).

[9] Hall M. D., Okabe M., Shen D.-W., Liang X.-J., Gottesman M. M., The Role of Cellular Accumulation in Determining Sensitivity to Platinum-Based Chemotherapy. Annu. Rev. Pharmacol. Toxicol. 48, 495–535 (2008). 17937596 10.1146/annurev.pharmtox.48.080907.180426

[10] Burger H., Loos W. J., Eechoute K., Verweij J., Mathijssen R. H. J., Wiemer E. A. C., Drug transporters of platinum-based anticancer agents and their clinical significance. Drug Resist. Updat. 14, 22–34 (2011). 21251871 10.1016/j.drup.2010.12.002

[11] Afratis N., Gialeli C., Nikitovic D., Tsegenidis T., Karousou E., Theocharis A. D., Pavão M. S., Tzanakakis G. N., Karamanos N. K., Glycosaminoglycans: key players in cancer cell biology and treatment. FEBS J. 279, 1177–1197 (2012). 22333131 10.1111/j.1742-4658.2012.08529.x

[12] Zhang F., Zhang Z., Linhardt R. J., in Handbook of Glycomics, Cummings R. D., Pierce J. M., Eds. (Academic Press, San Diego, 2010), pp. 59–80.

[13] Fuster M. M., Esko J. D., The sweet and sour of cancer: glycans as novel therapeutic targets.Nat. Rev. Cancer 5, 526–542 (2005). 16069816 10.1038/nrc1649

[14] Yip G. W., Smollich M., Götte M., Therapeutic value of glycosaminoglycans in cancer. Mol. Cancer Ther. 5, 2139–2148 (2006). 16985046 10.1158/1535-7163.MCT-06-0082

[15] Vallen M. J. E., van der Steen S. C. H. A., van Tilborg A. A. G., Massuger L. F. A. G., van Kuppevelt T. H., Sulfated sugars in the extracellular matrix orchestrate ovarian cancer development: ‘When sweet turns sour.’ Gynecol. Oncol. 135, 371–381 (2014). 25158037 10.1016/j.ygyno.2014.08.023

[16] Afratis N., Gialeli C., Nikitovic D., Tsegenidis T., Karousou E., Theocharis A. D., Pavão M. S., Tzanakakis G. N., Karamanos N. K., Glycosaminoglycans: key players in cancer cell biology and treatment. FEBS J. 279, 1177–1197 (2012). 22333131 10.1111/j.1742-4658.2012.08529.x

[17] Vallen M. J. E., Massuger L. F. A. G., ten Dam G. B., Bulten J., van Kuppevelt T. H., Highly sulfated chondroitin sulfates, a novel class of prognostic biomarkers in ovarian cancer tissue. Gynecol. Oncol. 127, 202–209 (2012). 22733095 10.1016/j.ygyno.2012.06.022

[18] Clausen T. M., Pereira M. A., Nakouzi N. A., Oo H. Z., Agerbæk M. Ø., Lee S., Ørum-Madsen M. S., Kristensen A. R., El-Naggar A., Grandgenett P. M., Grem J. L., Hollingsworth M. A., Holst P. J., Theander T., Sorensen P. H., Daugaard M., Salanti A., Oncofetal Chondroitin Sulfate Glycosaminoglycans Are Key Players in Integrin Signaling and Tumor Cell Motility. Mol. Cancer Res. 14, 1288–1299 (2016). 27655130 10.1158/1541-7786.MCR-16-0103 PMC5136311

[19] Berdiaki A., Neagu M., Giatagana E.-M., Kuskov A., Tsatsakis A. M., Tzanakakis G. N., Nikitovic D., Glycosaminoglycans: Carriers and Targets for Tailored Anti-Cancer Therapy. Biomolecules 11, 395 (2021). 33800172 10.3390/biom11030395 PMC8001210

[20] Lindahl U., Couchman J., Kimata K., Esko J. D., in Essentials of Glycobiology, Varki A., Cummings R. D., Esko J. D., Stanley P., Hart G. W., Aebi M., Darvill A. G., Kinoshita T., Packer N. H., Prestegard J. H., Schnaar R. L., Seeberger P. H., Eds. (Cold Spring Harbor Laboratory Press, Cold Spring Harbor (NY), 2015). 27010055

[21] Esko J. D., Prestegard J. H., Linhardt R. J., in Essentials of Glycobiology, Varki A., Cummings R. D., Esko J. D., Stanley P., Hart G. W., Aebi M., Darvill A. G., Kinoshita T., Packer N. H., Prestegard J. H., Schnaar R. L., Seeberger P. H., Eds. (Cold Spring Harbor Laboratory Press, Cold Spring Harbor (NY), 2015). 27010055

[22] Salanti A., Clausen T. M., Agerbæk M. Ø., Al Nakouzi N., Dahlbäck M., Oo H. Z., Lee S., Gustavsson T., Rich J. R., Hedberg B. J., Mao Y., Barington L., Pereira M. A., LoBello J., Endo M., Fazli L., Soden J., Wang C. K., Sander A. F., Dagil R., Thrane S., Holst P. J., Meng L., Favero F., Weiss G. J., Nielsen M. A., Freeth J., Nielsen T. O., Zaia J., Tran N. L., Trent J., Babcook J. S., Theander T. G., Sorensen P. H., Daugaard M., Targeting Human Cancer by a Glycosaminoglycan Binding Malaria Protein. Cancer Cell 28, 500–514 (2015). 26461094 10.1016/j.ccell.2015.09.003 PMC4790448

[23] Seiler R., Oo H. Z., Tortora D., Clausen T. M., Wang C. K., Kumar G., Pereira M. A., Ørum-Madsen M. S., Agerbæk M. Ø., Gustavsson T., Nordmaj M. A., Rich J. R., Lallous N., Fazli L., Lee S. S., Douglas J., Todenhöfer T., Esfandnia S., Battsogt D., Babcook J. S., Al-Nakouzi N., Crabb S. J., Moskalev I., Kiss B., Davicioni E., Thalmann G. N., Rennie P. S., Black P. C., Salanti A., Daugaard M., An Oncofetal Glycosaminoglycan Modification Provides Therapeutic Access to Cisplatin-resistant Bladder Cancer. Eur. Urol. 72, 142–150 (2017). 28408175 10.1016/j.eururo.2017.03.021

[24] Sand N. T., Petersen T. B., Bang-Christensen S. R., Ahrens T. D., Løppke C., Jørgensen A. M., Gustavsson T., Choudhary S., Theander T. G., Salanti A., Agerbæk M. Ø., Optimization of rVAR2-Based Isolation of Cancer Cells in Blood for Building a Robust Assay for Clinical Detection of Circulating Tumor Cells. Int. J. Mol. Sci. 21, 2401 (2020). 32244341 10.3390/ijms21072401 PMC7178266

[25] Spliid C. B., Toledo A. G., Sanderson P., Mao Y., Gatto F., Gustavsson T., Choudhary S., Saldanha A. L., Vogelsang R. P., Gögenur I., Theander T. G., Leach F. E., Amster I. J., Esko J. D., Salanti A., Clausen T. M., The specificity of the malarial VAR2CSA protein for chondroitin sulfate depends on 4-O-sulfation and ligand accessibility. J. Biol. Chem. 297 (2021), doi:10.1016/j.jbc.2021.101391. PMC8661001 34762909

[26] Oo H. Z., Lohinai Z., Khazamipour N., Lo J., Kumar G., Pihl J., Adomat H., Nabavi N., Behmanesh H., Zhai B., Dagil R., Choudhary S., Gustavsson T., Clausen T. M., Esko J. D., Allen J. W., Thompson M. A., Tran N. L., Moldvay J., Dome B., Salanti A., Al-Nakouzi N., Weiss G. J., Daugaard M., Oncofetal Chondroitin Sulfate Is a Highly Expressed Therapeutic Target in Non-Small Cell Lung Cancer. Cancers 13, 4489 (2021). 34503301 10.3390/cancers13174489 PMC8430715

[27] Vidal-Calvo E. E., Martin-Salazar A., Choudhary S., Dagil R., Raghavan S. S. R., Duvnjak L., Nordmaj M. A., Clausen T. M., Skafte A., Oberkofler J., Wang K., Agerbæk M. Ø., Løppke C., Jørgensen A. M., Ropac D., Mujollari J., Willis S., Garcias López A., Miller R. L., Karlsson R. T. G., Goerdeler F., Chen Y.-H., Colaço A. R., Wang Y., Lavstsen T., Martowicz A., Nelepcu I., Marzban M., Oo H. Z., Ørum-Madsen M. S., Wang Y., Nielsen M. A., Clausen H., Wierer M., Wolf D., Gögenur I., Theander T. G., Al-Nakouzi N., Gustavsson T., Daugaard M., Salanti A., Tumor-agnostic cancer therapy using antibodies targeting oncofetal chondroitin sulfate. Nat. Commun. 15, 7553 (2024). 39215044 10.1038/s41467-024-51781-0 PMC11364678

[28] Tang J., Zheng Q., Wang Q., Zhao Y., Ananthanarayanan P., Reina C., Šabanović B., Jiang K., Yang M.-H., Meny C. C., Wang H., Agerbaek M. Ø., Clausen T. M., Gustavsson T., Wen C., Borghi F., Mellano A., Fenocchio E., Gregorc V., Sapino A., Theander T. G., Fu D., Aicher A., Salanti A., Shen B., Heeschen C., CTC-derived pancreatic cancer models serve as research tools and are suitable for precision medicine approaches. Cell Rep. Med. 5, 101692 (2024). 39163864 10.1016/j.xcrm.2024.101692 PMC11524981

[29] Løppke C., Ugleholdt R., Secher C. F., Sand N. T., Mujollari J., Gustavsson T., Dagil R., Theander T. G., Salanti A., Rohrberg K. S., Agerbæk M. Ø., Multi-cancer detection of circulating tumor cells by targeting oncofetal chondroitin sulfate. NPJ Precis. Oncol. 9, 144 (2025). 40379789 10.1038/s41698-025-00936-3 PMC12084527

[30] Enciso Martinez A., Løppke C., Koene J. J., Ebbelaar J., van der Geest M., Los M., Boerrigter E. P. E. M., Dagil R., Gustavsson T., Vidal Calvo E. E., van Leeuwen T. G., Koning R. I., van Es N., Salanti A., van der Pol E., Nieuwland R., Agerbæk M. Ø., ten Dijke P., Targeting Oncofoetal Chondroitin Sulphate Allows Identification of Tumour Derived Extracellular Vesicles. J. Extracell. Vesicles 14, e70106 (2025). 40527727 10.1002/jev2.70106 PMC12173530

[31] Zhao Y., Wen C., Wang Q., Qing Y., Tondi S., Reina C., Šabanović B., Chang C. Y., Lai C., Wang H., Agerbaek M. Ø., Clausen T. M., Gustavsson T., Theander T. G., Salanti A., Meny C. C., Shen B., Aicher A., Tang J., Heeschen C., Use of the Malaria Protein VAR2CSA for the Detection of Small Extracellular Vesicles to Diagnose Adenocarcinoma. J. Extracell. Vesicles 14, e70067 (2025). 40241173 10.1002/jev2.70067 PMC12003099

[32] Silva H., Frézard F., Peterson E. J., Kabolizadeh P., Ryan J. J., Farrell N. P., Heparan Sulfate Proteoglycan-Mediated Entry Pathway for Charged Tri-Platinum Compounds: Differential Cellular Accumulation Mechanisms for Platinum. Mol. Pharm. 9, 1795–1802 (2012). 22494465 10.1021/mp300098t PMC3367083

[33] Peterson E. J., Daniel A. G., Katner S. J., Bohlmann L., Chang C.-W., Bezos A., Parish C. R., von Itzstein M., Berners-Price S. J., Farrell N. P., Antiangiogenic platinum through glycan targeting. Chem. Sci. 8, 241–252 (2017). 28451171 10.1039/c6sc02515c PMC5355868

[34] Hampton J. D., Peterson E. J., Katner S. J., Turner T. H., Alzubi M. A., Harrell J. C., Dozmorov M. G., Turner J. B. M., Gigliotti P. J., Kraskauskiene V., Shende M., Idowu M. O., Puchallapalli M., Hu B., Litovchick L., Katsuta E., Takabe K., Farrell N. P., Koblinski J. E., Exploitation of sulfated glycosaminoglycan status for precision medicine of Triplatin in triple-negative breast cancer. Mol. Cancer Ther. (2021), doi:10.1158/1535-7163.MCT-20-0969. PMC8828673 34815360

[35] Lawrence R., Olson S. K., Steele R. E., Wang L., Warrior R., Cummings R. D., Esko J. D., Evolutionary Differences in Glycosaminoglycan Fine Structure Detected by Quantitative Glycan Reductive Isotope Labeling. J. Biol. Chem. 283, 33674–33684 (2008). 18818196 10.1074/jbc.M804288200 PMC2586254

[36] Farrell N. P., Gorle A. K., Peterson E. J., Berners-Price S. J., in Metallo-Drugs: Development and Action of Anticancer Agents, (De Gruyter, 2018), pp. 109–140.

[37] Katner S. J., Ginsburg E. P., Hampton J. D., Peterson E. J., Koblinski J. E., Farrell N. P., A Comparison of Di- and Trinuclear Platinum Complexes Interacting with Glycosaminoglycans for Targeted Chemotherapy. ACS Med. Chem. Lett. 14, 1224–1230 (2023). 37736178 10.1021/acsmedchemlett.3c00244 PMC10510529

[38] Rottenberg S., Disler C., Perego P., The rediscovery of platinum-based cancer therapy. Nat. Rev. Cancer 21, 37–50 (2021). 33128031 10.1038/s41568-020-00308-y

[39] Gorle A. K., Berners-Price S. J., Farrell N. P., Biological relevance of interaction of platinum drugs with O-donor ligands. Inorganica Chim. Acta 495, 118974 (2019). 31354168 10.1016/j.ica.2019.118974 PMC6660021

[40] Skeltved N., Nordmaj M. A., Berendtsen N. T., Dagil R., Stormer E. M. R., Al-Nakouzi N., Jiang K., Aicher A., Heeschen C., Gustavsson T., Choudhary S., Gögenur I., Christensen J. P., Theander T. G., Daugaard M., Salanti A., Nielsen M. A., Bispecific T cell-engager targeting oncofetal chondroitin sulfate induces complete tumor regression and protective immune memory in mice. J. Exp. Clin. Cancer Res. CR 42, 106 (2023). 37118819 10.1186/s13046-023-02655-8 PMC10142489

[41] Nordmaj M. A., Roberts M. E., Sachse E. S., Dagil R., Andersen A. P., Skeltved N., Grunddal K. V., Erdoğan S. M., Choudhary S., Gustsavsson T., Ørum-Madsen M. S., Moskalev I., Tian W., Yang Z., Clausen T. M., Theander T. G., Daugaard M., Nielsen M. A., Salanti A., Development of a bispecific immune engager using a recombinant malaria protein. Cell Death Dis. 12, 353 (2021). 33824272 10.1038/s41419-021-03611-0 PMC8024270

[42] Qu Y., Kipping R. G., Farrell N. P., Solution studies on DNA interactions of substitution-inert platinum complexes mediated via the phosphate clamp. Dalton Trans. 44, 3563–3572 (2015). 25524170 10.1039/c4dt03237c PMC4323935

[43] Farrell N, Spinelli S., Dinuclear and Trinuclear Platinum Anticancer Agents. (Royal Society of Chemistry, Uses of Inorganic Chemistry in Medicine., 1999).

[44] Narimatsu Y., Joshi H. J., Yang Z., Gomes C., Chen Y.-H., Lorenzetti F. C., Furukawa S., Schjoldager K. T., Hansen L., Clausen H., Bennett E. P., Wandall H. H., A validated gRNA library for CRISPR/Cas9 targeting of the human glycosyltransferase genome. Glycobiology 28, 295–305 (2018). 29315387 10.1093/glycob/cwx101

