# Supplementary material for: Glycan Profiling Identifies Chondroitin-4-sulfate as a Biomarker for Platinum Response and Therapeutic Target in Ovarian Cancer
Source: bioRxiv. 2025 Oct 7:2025.10.06.675352. Preprint. [Version 1] doi: 10.1101/2025.10.06.675352 (PMC12632365; doi:10.1101/2025.10.06.675352)
Supplement: Supplement 1 [file media-1.docx]

**Fig. S1 GRIL-MS disaccharide analysis of glycosaminoglycans**. **(A)** Overview of the workflow for isolation, purification, and reductive isotopic labeling for MS analysis. **(B)** The extracted ion chromatogram (XIC) for each of a mixture of eight HS disaccharide standards. **(C)** The XIC for free molecular ion for [13C6] aniline-labeled HS disaccharide, D0A0, [M-H]−1 (*m*/*z* = 461). **(D)** Sensitivity and linear range for HS species (D0A0) shown as a correlation between picomole (pmol) amount and relative mass abundance. **(E)** The XIC for each of a mixture of seven CS/DS disaccharide standards. **(F)** XIC for the free molecular ion for [13C6] aniline-labeled CS/DS disaccharide, D0a0, [M-H]−1 (*m*/*z* = 461). **(G)** Sensitivity and linear range for CS/DS species (D0a0) are shown as a correlation between the picomole amount and relative mass abundance.


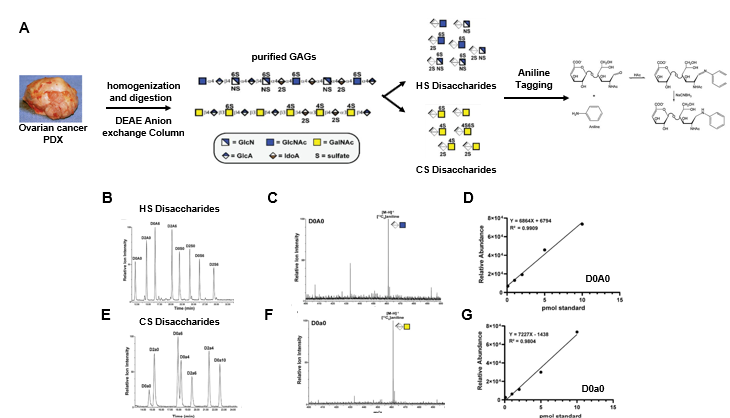

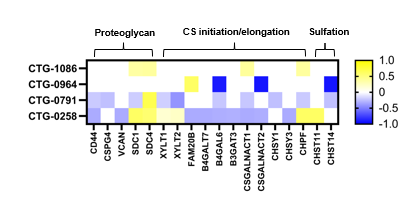


**Fig. S2 Bulk RNAseq analysis of genes involved in CS proteoglycan, initiation/elongation and sulfation**. Bulk RNA-seq analysis (TPM, log₂-transformed) provided by Champions Oncology comparing gene expression across four ovarian cancer PDX models (CTG-1086, CTG-0964, CTG-0791, CTG-0258). The heatmap displays relative expression of genes involved in proteoglycan core proteins, chondroitin sulfate (CS) chain initiation/elongation, and sulfation. Yellow indicates higher expression and blue indicates lower expression relative to the mean.


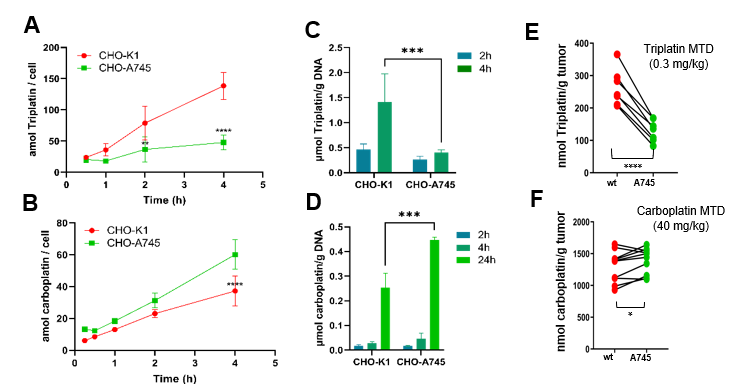


**Fig. S3 Effects of GAG levels on platinum cellular uptake, DNA platination, and tumor accumulation. AB**. Cellular platinum uptake in CHO-K1 (control) and CHO-A745 GAG-deficient cells treated with 10 µM Triplatin or 100 µM carboplatin. Platinum content was measured by inductively coupled plasma mass spectroscopy (ICP-MS) and normalized by number of cells. ****p <0.0001, student t-test. **C, D.** Levels of Pt-DNA adducts in K1 and A745 cells treated with 10 µM Triplatin or 100 µM carboplatin. Platinum content in isolated DNA was measured by ICP-MS and normalized DNA quantity. ***p <0.001, ANOVA **E, F.** NSG mice were injected with K1 (left flank) and A745 (right flank) tumor cells s.c. and tumors were allowed to grow to ~150 mm^3^. Mice (n=5) were treated i.p with Triplatin or carboplatin. Tumors were harvested after 24h, digested in acid, and the amount of platinum quantified by ICP-MS analysis. *p<0.05, ****p <0.0001, paired student t-test.


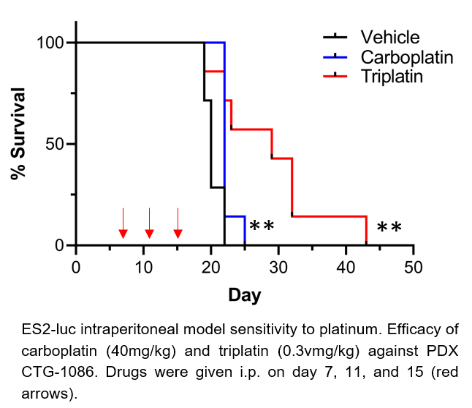


**Fig. S4 Increased survival in ES2-luc mouse model treated with Triplatin.** Survival analysis of ES-2-luc orthotopic model treated with carboplatin (40mg/kg) and Triplatin (0.3vmg/kg) given i.p. on day 7, 11, and 15 (red arrows). **p<0.01


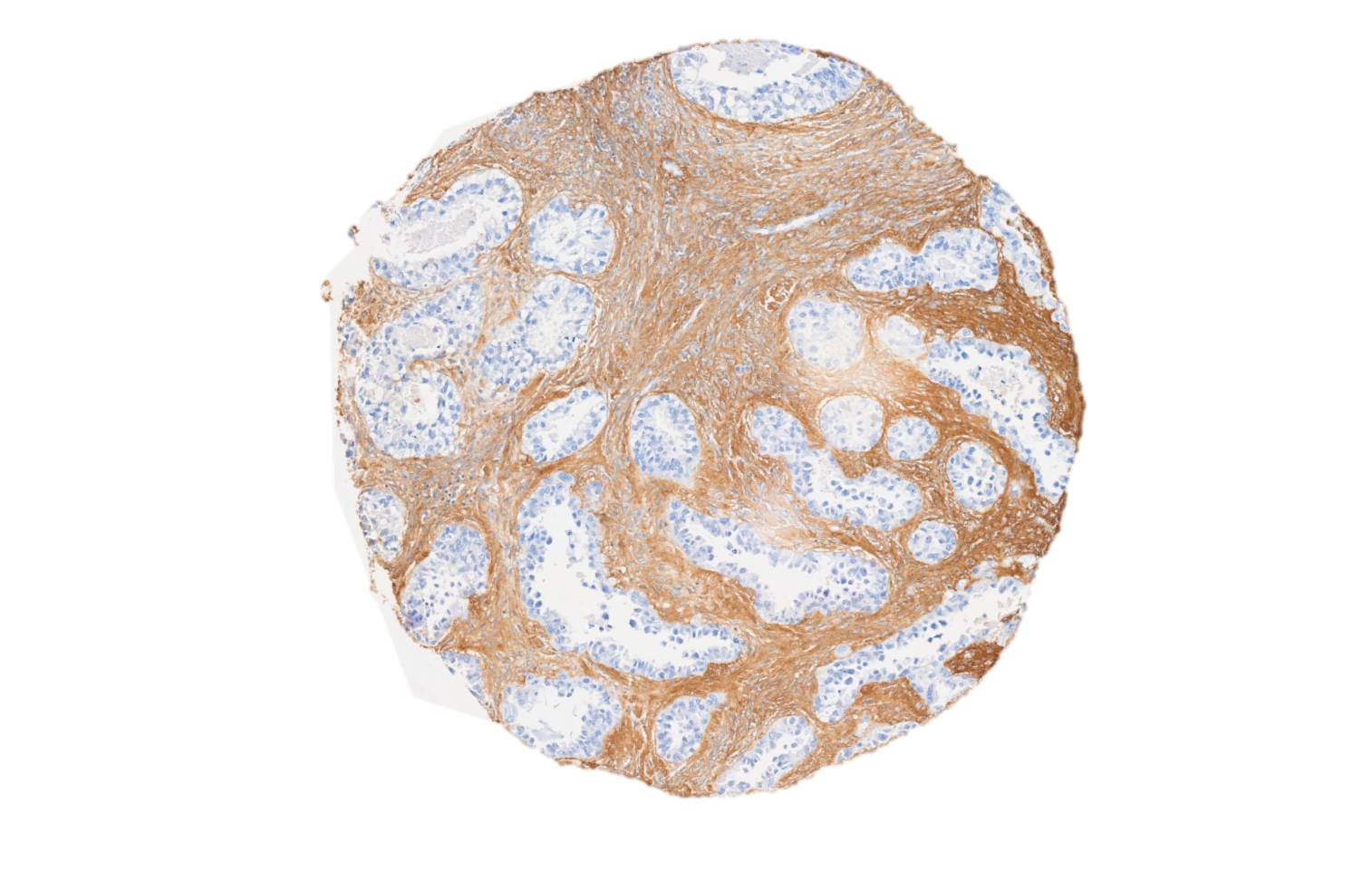

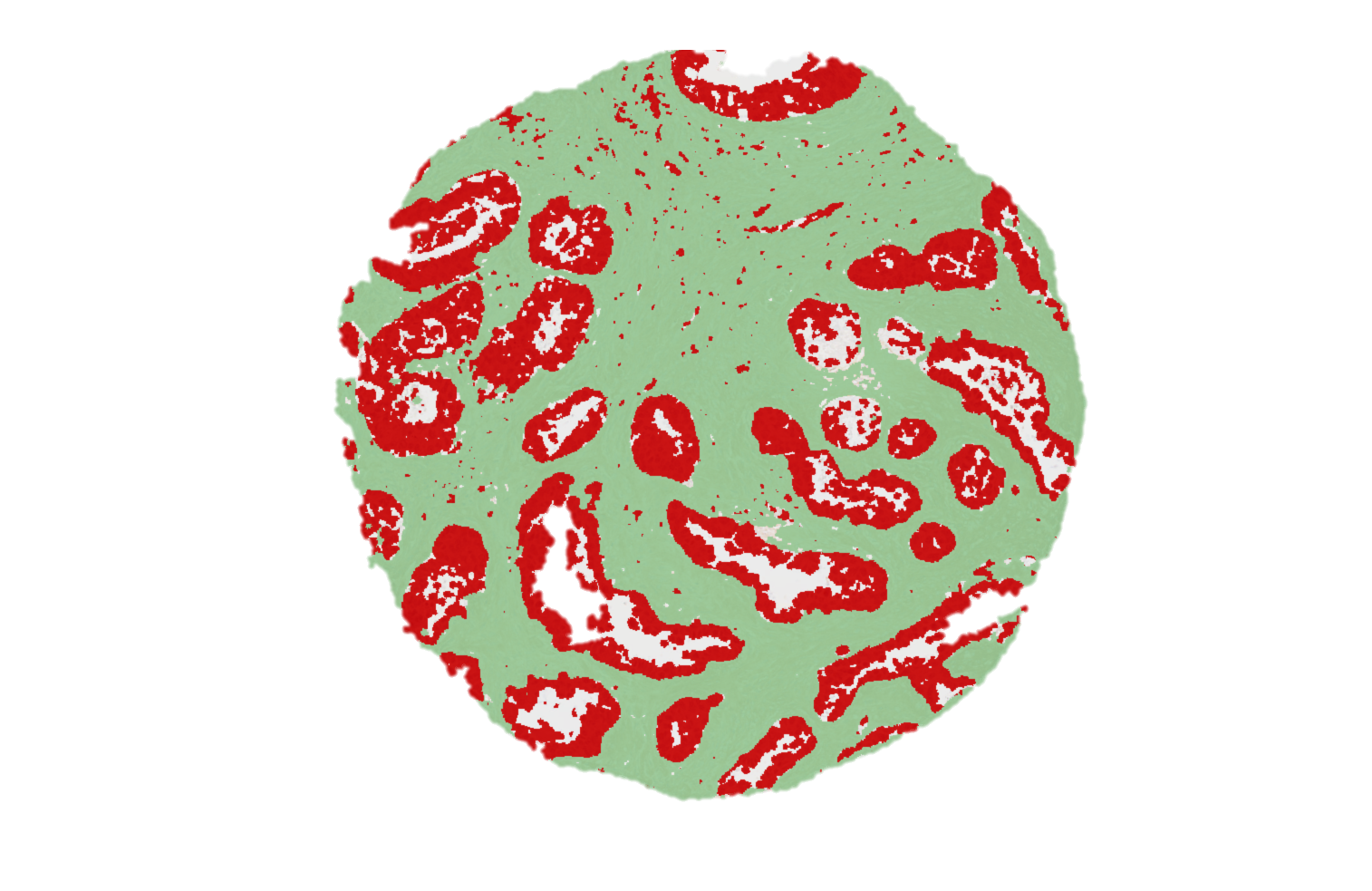

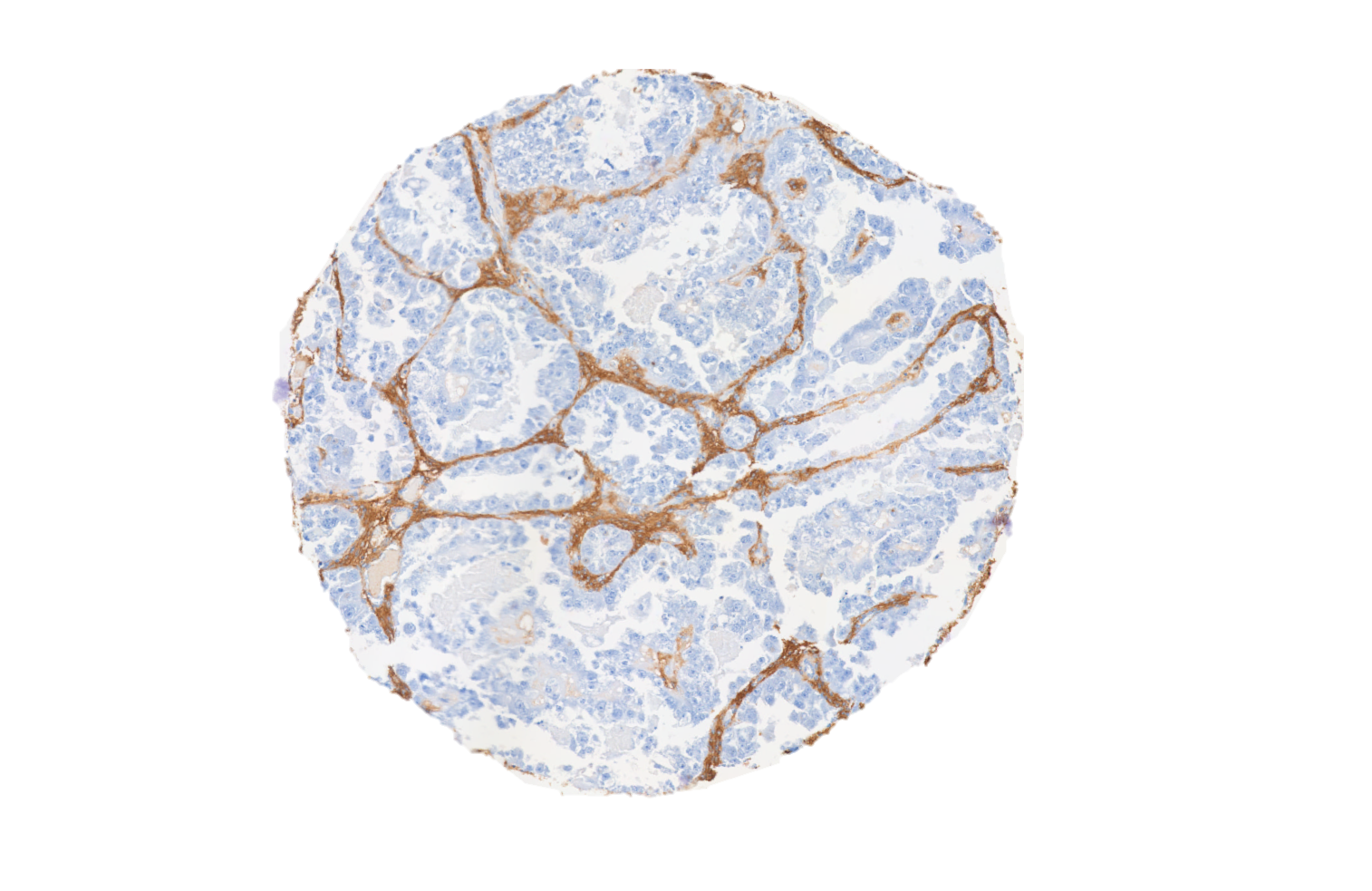

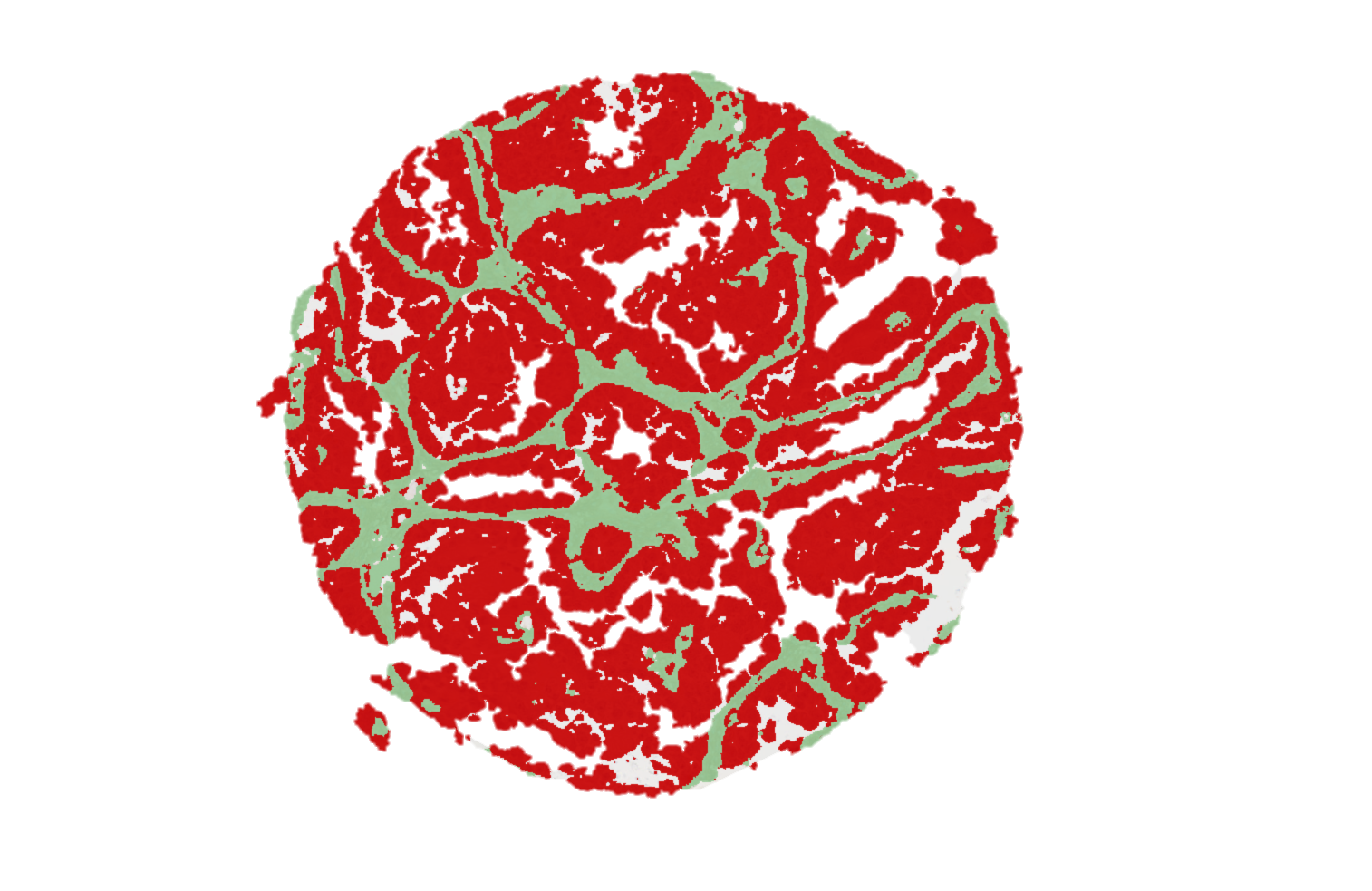


**A**

**B**

**C**

**D**

**E**

**F**


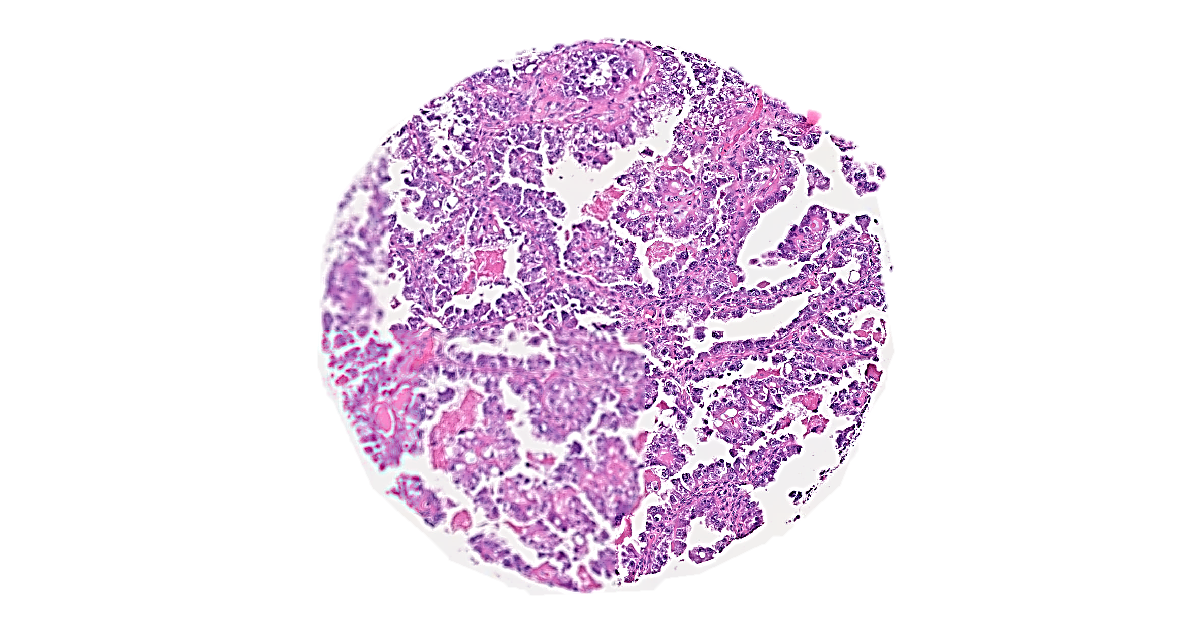

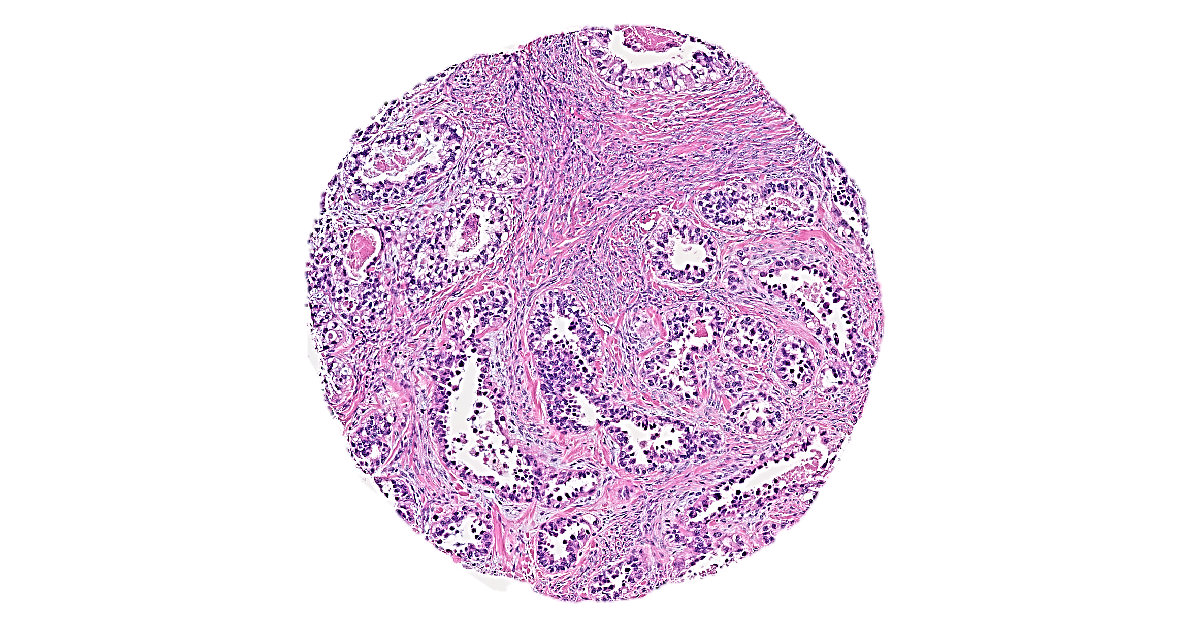


TSR=0.59

TSR=6.79


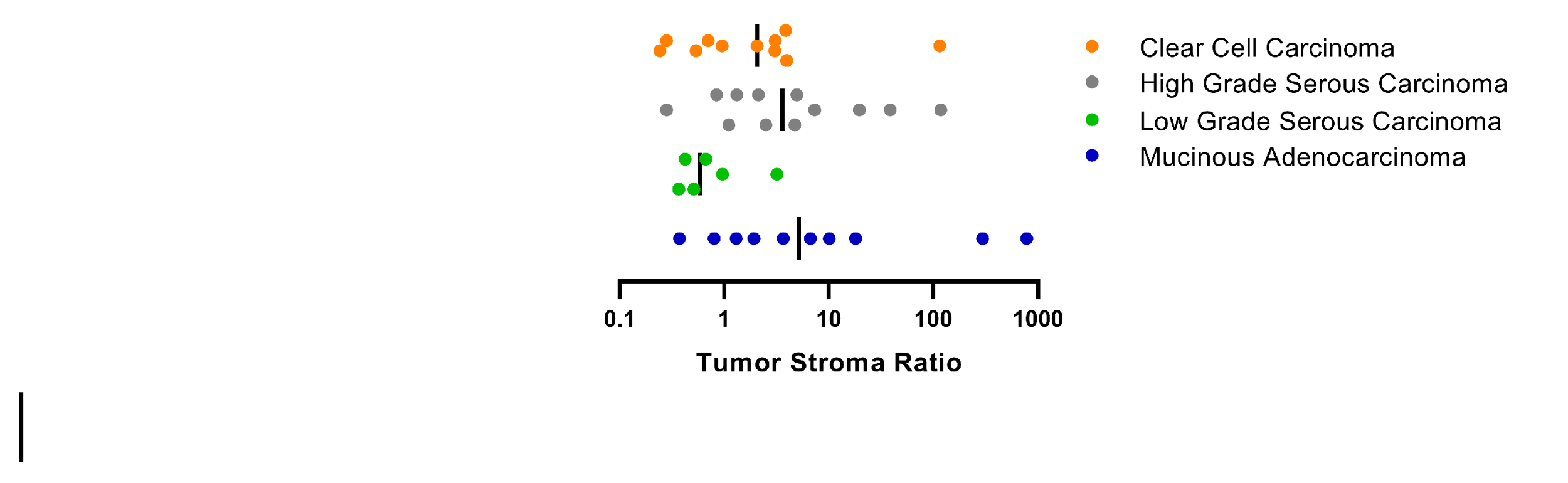


**G**

**Fig. S5. C4S staining colocalizes with tumor stroma.** Images display representative OC tumors with high stroma **(A-C)** and low stroma **(D-F)** comparing matched H&E staining **(A,D)**, rVAR2-V5 staining **(B,E)** and segmented images of tumor epithelium (red) and stroma (green) **(C,F)**. Using the pixel classifier algorithm in QuPath, segmented images were used to calculate the tumor stroma ratio (TSR). TMA shows 40% (16/40) with TSR<1.0 and 60% (26/40) with TSR > 1.0. **(G)** Distribution of TSR in various subtypes of ovarian cancer. Stromal-rich tumors (TSR<1.0); 45% CCC (5/11), 17% HGSC (2/12), 83% LGSC (5/6), 20% MAC (2/10). Tumor epithelium and stroma segmented using the pixel classifier algorithm in QuPath based on rVAR2 staining. *p<0.01, Fisher’s exact test. TMA CHTN OvCa2 provided by UVA


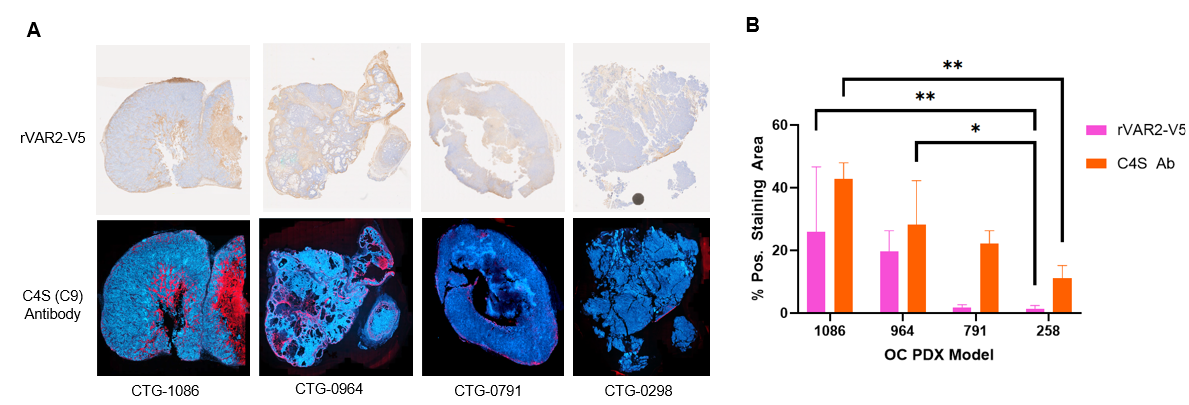


**Fig. S6. Correlation of rVAR2-V5 and C4S (C9) antibody staining. (A)** Representative ovarian cancer PDX tumors (CTG-1086, CTG-0964, CTG-0791, CTG-0298) stained with rVAR2-V5 (top row) or C4S (C9) antibody (bottom row).**(B)** Quantification of percent positive staining area calculated using QuPath. Bars represent mean ± SD of two replicate tumor sections per model. Statistical significance determined by one-way ANOVA with Tukey’s post-hoc test (* p < 0.05, ** p < 0.01).


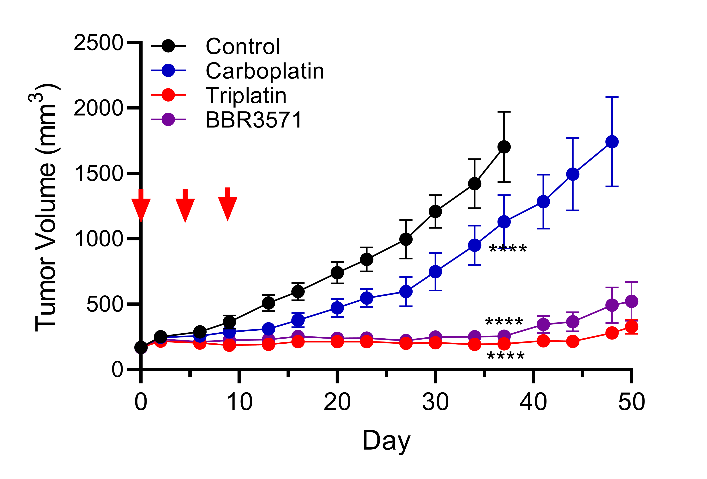

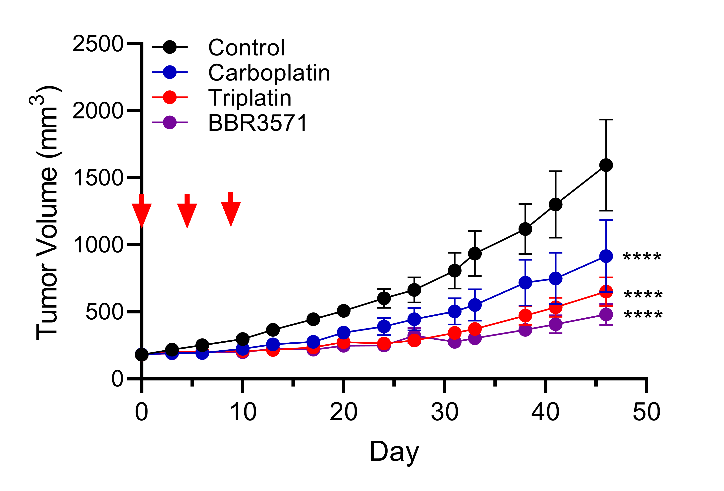

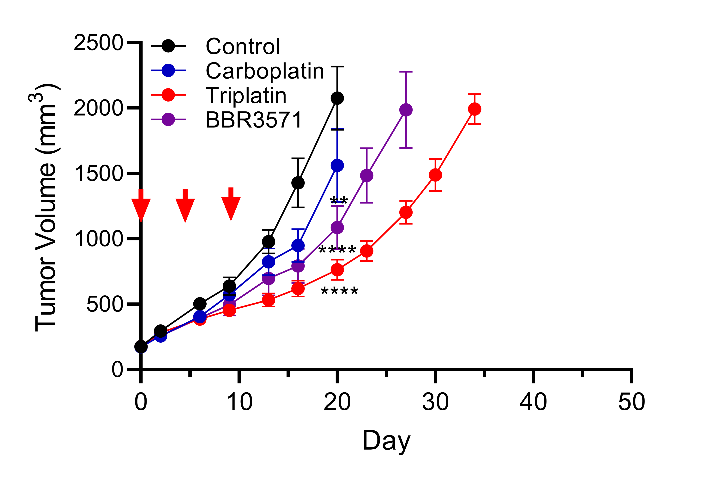

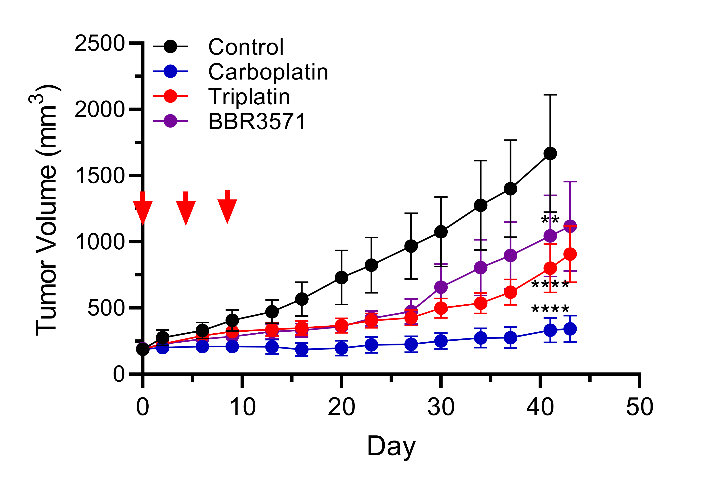


CTG-1086

CTG-0964

CTG-0791

CTG-0258

**A**

**B**

**C**

**D**

**Fig. S7 Similar sensitivity of OC PDX models to Triplatin and BR3571. P**DX models were treated i.p. with carboplatin (40 mg/kg), BBR3571 (0.3 mg/kg) or Triplatin (0.3 mg/kg) on days 0, 4 and 8.
